# Effectiveness of High Power Laser Therapy on Pain and Isokinetic Peak Torque in Athletes with Proximal Hamstring Tendinopathy: A Randomized Trial

**DOI:** 10.1155/2022/4133883

**Published:** 2022-05-20

**Authors:** Sachin Verma, Vandana Esht, Aksh Chahal, Gaurav Kapoor, Sorabh Sharma, Ahmad H. Alghadir, Masood Khan, Faizan Z. Kashoo, Mohammad A. Shaphe

**Affiliations:** ^1^Maharishi Markandeshwar Institute of Physiotherapy and Rehabilitation (Deemed to be University), Mullana, Ambala, Haryana 133207, India; ^2^Chitkara School of Health Sciences, Chitkara University, Punjab, India; ^3^KYNA Physiotherapy, Patiala, Punjab, India; ^4^Department of Rehabilitation Sciences, College of Applied Medical Sciences, King Saud University, Riyadh 11433, Saudi Arabia; ^5^Department of Physical Therapy and Health Rehabilitation, College of Applied Medical Sciences, Majmaah University, Majmaah, 11952, Saudi Arabia; ^6^Department of Physical Therapy, College of Applied Medical Science, Jazan University, Jazan, 45142, Saudi Arabia

## Abstract

Athletes such as long-distance runners, sprinters, hockey, and/or football players may have proximal hamstring tendinopathy (PHT). Laser therapy has been shown to be effective in tendinopathies. High power laser therapy (HPLT) is used for the treatment of several musculoskeletal conditions; however, its efficacy on PHT has not been investigated. This study is aimed at examining the effects of HPLT on pain and isokinetic peak torque (IPT) in athletes with PHT. The two-arm comparative pretest-posttest experimental design was used with random allocation of 36 athletes aged 18-35 years into two groups (experimental and conventional group). The experimental group included the application of HPLT for 3 weeks. The conventional group included treatment with a conventional physiotherapy program including ultrasound therapy, moist heat pack, and home exercises for a total of 3 weeks. Pain and IPT of the hamstring muscle were measured before and after the application of the intervention. Pain score decreased, and IPT increased significantly (*p* < 0.05) after application of HPLT, by 61.26% and 13.18%, respectively. In the conventional group, a significant difference (*p* < 0.05) was observed in pain scores only, which decreased by 41.14%. No significant difference (*p* > 0.05) was observed in IPT in the conventional group. When HPLT was compared with conventional physiotherapy, a significant difference was found in pain scores only. HPLT for 3 weeks was found to be effective in improving pain in athletes with PHT. However, no significant difference was found between HPLT and conventional physiotherapy (US, moist heat, and home exercises) in improving the IPT of the hamstring muscle.

## 1. Introduction

Proximal hamstring tendinopathy (PHT) is tendinopathy of the semimembranosus and/or biceps femoris/semitendinosus complex [1]. This condition is common among middle and long-distance runners, athletes who perform more sagittal plane activities (e.g. sprinters), and individuals including nonathletes, who routinely perform activities like leaning forward, sitting for long periods, excessive static stretching, squatting, lunging, or changing the direction of running [2–4]. These activities compressively load the hamstring tendon at its proximal attachment. The main symptom of PHT is deep localized pain in the lower gluteal region near the ischial tuberosity, which may or may not radiate to the posterior thigh [5]. This pain often worsens during or after sitting, squatting, lunging, or running [4]. This condition may be present unilaterally or bilaterally [4].

Surgery is indicated for recalcitrant cases, and most cases are treated conservatively. Physical therapy treatment of PHT focuses on activity modification, effective tendon loading including eccentric training, addressing contributing biomechanical deficiencies, and electrotherapy including laser therapy. Two types of laser therapy are used as a part of physical therapy management: low power laser therapy (LPLT) which has an output power of less than 0.5 watts and high power laser therapy (HPLT) which has an output power of 0.5 watts or greater.

Previous studies have shown the efficacy of laser therapy in the treatment of tendinopathy. The study by Stergioulas et al. [6] showed that LPLT when added to an eccentric exercises regimen speeds up clinical recovery in patients with chronic Achilles tendinopathy. HPLT produces more powerful beams (power > 0.5 watts) and has longer laser emission intervals and shorter laser emission time in comparison to LPLT; thus, the deeper areas can be irradiated in a short time with HPLT [7, 8]. HPLT also creates heat on the skin surface due to its higher power density.

A recent systematic review by Taradaj et al. indicated the effectiveness of HPLT in decreasing musculoskeletal pain [9]. Other studies also showed that HPLT affects the repair of diabetic foot ulcer trauma [10], gonitis [11], shoulder pain [8], chronic low back pain [12, 13], chronic neck pain [14], and pain in the knee osteoarthritis [15, 16]. HPLT can also remove exudates through increased metabolism and blood circulation, thus helping in the quick absorption of edema [8]. Few physiological changes occur in tissues as a result of HPLT which do not occur in the case of a conventional physiotherapy program for tendinopathy including ultrasound therapy, moist heat pack, and eccentric hamstring exercises. HPLT radiation causes slow and small light absorption by the chromophores. The absorption by chromophores occurs with diffuse light in all directions, not with concentrated light, which is called the scattering phenomenon. This leads to the phenomenon of tissue stimulation (photobiology effects) and an increase in the mitochondrial oxidative reaction and DNA, RNA, or adenosine triphosphate production (photochemistry effects) [17].

To the best of our knowledge, no study has compared the effects of HPLT with conventional physiotherapy programs in athletes suffering from PHT. Therefore, this study is aimed at assessing the effects of HPLT on pain and isokinetic peak torque (IPT) of hamstring muscle in PHT patients. We hypothesized that HPLT is effective in reducing pain and improving the IPT of hamstring muscle in comparison to conventional physiotherapy program in patients with PHT. In the present study, the conventional physiotherapy program included ultrasound therapy [18], moist heat pack [19], and eccentric hamstring exercises [20].

## 2. Materials and Methods

### 2.1. Study Design

We used a two-arm parallel pretest-posttest experimental research design with random allocation of subjects into two groups (experimental and conventional group).

### 2.2. Participants

#### 2.2.1. Sample Size Calculation

**Figure 1:**
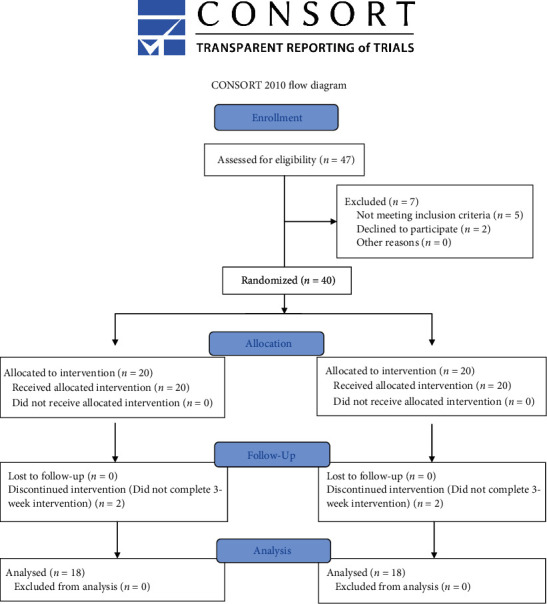
Consolidated Standards of Reporting Trials (CONSORT) flow chart of the study showing the recruitment of participants.

Before conducting the study, the sample size was calculated using the software G∗Power 3.1.9.4. The pain score data from the study of Elsodany et al. [21], who used high intensity laser therapy on patients with rotator cuff tendinopathy, was used to calculate the effect size. Based on *α* = 0.05, power (1‐*β*) = 0.95, and effect size *d* = 3.89, the minimum sample size was calculated to be 5 (including 12% drop out) in each group. Therefore, due to the availability of patients, a total of 36 participants aged 18-35 years were recruited in the present study (Table 1) (Figure 1).

#### 2.2.2. Inclusion and Exclusion Criteria

The selected participants were diagnosed with PHT by a consultant physiotherapist. They were athletes, who took part in national level competitive track and field events more than once, with subacute onset of the buttock or posterior thigh pain for less than a year, tenderness in the ischial tuberosity, tightness deep of the hamstring muscle, deeper hip flexion such as squatting or sitting for long periods, repeated knee extension, and resisted knee flexion increased their pain. Other differential diagnoses like radiation due to lumbosacral radiculopathy, piriformis syndrome, or ischiofemoral impingement were ruled out by an expert physiotherapist. Participants who had a recent history of trauma to the posterior thigh, a musculoskeletal disorder or deformity of the ipsilateral lower extremity, lumbar prolapsed intervertebral disc, history of or currently taking pain medications, cardiovascular diseases, malignant tumor in the lower extremity, phlebitis, blood disorders, or tattoo over or around the area of treatment were excluded from the study because these conditions will affect the application of laser therapy or exercise of hamstring muscles. The repeated movement of the lumbar spine, sacroiliac joint provocation tests, SLR, and slump test did not aggravate their pain.

#### 2.2.3. Randomization of Participants and Blinding

The selected participants were randomly assigned to an experimental and a conventional group using the lottery method and http://randomization.com/ website with 18 participants in each group. The participants and outcome assessor were kept blind to the allocation.

#### 2.2.4. Setting, Ethical Statement, Clinical Trial Registration, and Informed Consent

The study was carried out in the clinical setting of the University. The present study conformed to the “The Code of Ethics of the World Medical Association (Declaration of Helsinki)” and was approved by the ethical committee of the Institutional Review Board (file ID: RRC-2021-07; date of approval: 9 March 2021). This study had been retrospectively registered on Protocol Registration and Results System (PRS) clinicaltrials.gov (ID: NCT05100394) on 31^st^ October 2021. The risks and benefits of the study were discussed with each participant before the start of the study, and informed consent was obtained from all participants involved in the study.

### 2.3. Outcome Measures

The following are the outcome measures:

- Isokinetic peak torque of the hamstring muscle, assessed using an isokinetic dynamometer
- Pain, assessed using the NPRS (Numeric Pain Rating Scale) score

### 2.4. Instrumentation

The following are the instruments used:

- LASER equipment (LiteCure, USA) [22]
- Isokinetic dynamometer (Easy Tech Biomed, India) [23]
- Ultrasound therapy equipment (Physiocare, India) [24]
- Moist heat pack [25]

### 2.5. Study Protocol

The study consisted of three phases:

#### 2.5.1. Preintervention Assessment

Baseline NPRS (Numeric Pain Rating Scale) and IPT of hamstring muscle were measured before the start of the intervention. (i) The Numeric Pain Rating Scale (NPRS) is a subjective measure in which participants are asked to rate their pain on a scale of 0–10, where 0 represents “no pain” and 10 represents “worst pain imaginable” [26, 27]. The participants were asked to rate their pain on the NPRS scale. (ii) In the IPT of hamstring muscle, participants were asked to sit on the isokinetic dynamometer chair. Shoulders, chest, and hips were strapped to prevent unnecessary movements. The cuff of the dynamometer arm was attached near the ankle of the ipsilateral side. The back seat of the dynamometer was tilted 75-85° backward. The angle on the dynamometer was set from 0° (full knee extension) to 90° knee flexion, and the speed was selected at 90°/s. Before taking the readings for baseline measurement, each participant was asked to practice the movement thrice with submaximal effort. The participants were then asked to bend the knee with maximum effort. A total of three measurements were taken, and the highest reading was used for data analysis [28].

#### 2.5.2. Intervention

- Experimental group: the participants were made to lie prone, and the area around the ischial tuberosity was uncovered. Participants were asked to remove the excess hairs if present. HPLT was administered as monotherapy, in the area of ischial tuberosity where the hamstring tendons originate. The following parameters were used in laser equipment: average output power: 5 watts, dosage: 50 joules/cm^2^, laser wavelength: 980/810 nm, total treatment area: 6 cm × 6 cm = 36 cm^2^, and total energy: 50 × 36 = 1800 joules. Depending upon the total area to be treated and average output power and the total energy to be delivered, the total treatment time was calculated to be 6 minutes [29]. Therefore, HPLT was applied in continuous mode for a total of 6 minutes. HPLT was administered 3 days a week for a total of 3 weeks [30].
- Conventional group: conventional physiotherapy treatment was administered that included ultrasound therapy (continuous mode, 1 MHz, 2 W/cm^2^ for 5 minutes) in the area of the ischial tuberosity, moist heat packs (10 minutes) [31] over the ipsilateral buttock and posterior thigh region, and home exercises. The US and moist heat pack were applied in the prone position. Home exercises included Nordic hamstring exercise (eccentric hamstring contractions) [1]—2 sets of 5 repetitions. At home, participants were asked to stabilize their feet either under furniture/immovable objects or ask someone to hold their feet firmly. Then, they have to slowly lower their body from a vertical position towards the ground while maintaining a straight line from knees to head. Participants were allowed to use their hands to catch themselves if they cannot control the body movement from their knees. This treatment regimen was also administered 3 days a week for a total of 3 weeks [5].

#### 2.5.3. Postintervention Assessment

After completion of the intervention, the NPRS score and IPT of the hamstring muscle were again measured similarly to the preintervention assessment.

### 2.6. Data Analysis

The baseline values of NPRS and IPT were compared between both groups using the independent sample *t*-test, which revealed no significant difference (*p* < 0.05); therefore, both groups were comparable for both variables. The Shapiro-Wilk normality test was performed to assess the normal distribution of the baseline NPRS and IPT values. The Shapiro-Wilk test revealed that the distribution of baseline NPRS values was not normal (*p* < 0.05); therefore, for further with-in and between-group comparison, nonparametric tests were used. Wilcoxon's signed-rank test and Mann–Whitney *U* test were performed for within and between-group comparison, respectively. The confidence interval was set at 95%; *p* < 0.05 was considered significant (Table 2).

## 3. Results and Discussion

### 3.1. Within-Group (Wilcoxon's Signed Rank Test) Analysis

#### 3.1.1. For the Experimental Group

There was a significant difference (*p* < 0.05) in both variables (NPRS scores and IPT values) after the application of the intervention. NPRS scores decreased by 61.26%, and IPT increased by 13.18% after HPLT.

#### 3.1.2. For the Conventional Group

There was a significant difference (*p* < 0.05) in the NPRS scores after application of intervention; however, for IPT there was no significant difference (*p* > 0.05). NPRS scores decreased by 41.14%, and IPT increased marginally by 1.49%.

### 3.2. Between-Group (Mann–Whitney *U* Test) Analysis

#### 3.2.1. For Post_NPRS Scores

There was a significant difference (*p* ≤ 0.001) in post_NPRS scores between both groups.

#### 3.2.2. For Post_IPT Values

There were no significant differences (*p* = 0.131) for post_IPT values between the two groups.

The results of the present study revealed that HPLT is effective in improving pain scores and hamstring IPT in athletes with PHT; however, compared to the conventional group (US, moist heat, and home exercises), a significant difference was found only in NPRS scores. With the application of HPLT, NPRS scores decreased and IPT increased. Conventional physiotherapy (US, moist heat, and home exercises), treatment also decreased NPRS scores; however, IPT remained unchanged. HPLT was more effective in reducing pain than the conventional physiotherapy program. With conventional physiotherapy treatment, no improvement in IPT was observed, perhaps because patients performed eccentric Nordic hamstring exercises, which may have put a strain on the hamstring tendons and prevented the muscle from being unloaded.

In earlier studies, laser therapy was found to be effective in relieving pain associated with several conditions such as knee injuries, shoulder pain, fibromyalgia, chronic arthritis, carpal tunnel syndrome, and tendonitis [32, 33]. A systematic review reported that acute neck pain decreased immediately after laser therapy and up to 22 weeks after complete treatment [34].

At different levels, several physiological effects of laser therapy have been reported that produce analgesic effects. At the tissue level, laser causes reduction of histamine and bradykinin release from the injured tissues [35], increases the pain thresholds [36], and reduces the secretion of substance P from peripheral nociceptors [37]. Laser therapy slows the transmission of pain signals by decreasing the conduction velocity and increasing the latency of sensory nerves, which in turn inhibit A*δ*- and C fiber transmission [38]. Furthermore, laser treatment inhibits pain centrally, by increasing the secretion of endogenous opioids (*β*-endorphin) [39]. Specifically, HPLT application has been found to assist in pain relief [15], recovery from nerve paralysis [40], and wound repair [41]. It was also used to provide relief from shoulder pain [8], low back pain [12], and chronic ankle pain [42]. HPLT has not been found to reduce inflammation, but it had an analgesic effect on nerve endings [43, 44].

The analgesic effects of HPLT obtained in the present study can be explained by two mechanisms. If it is used in pulse mode, it has analgesic effects on nerve endings [43, 44]. This mode of application inhibits nociceptive stimulation and produces low heat. If a continuous mode is used, then photochemical and photothermic effects are produced in deeper tissues. These effects increase vascular permeability, blood flow, and cell metabolism which result in the washing out of cytokines that justifies pain reduction [45].

In our study, HPLT resulted in improvement in IPT of the hamstring muscle. Not many studies have examined the effects of HPLT on muscle strength. A study by Santamato et al. reported improved muscle strength of shoulder joints affected with subacromial impingement syndrome after application of HPLT [8]. Some studies have reported no significant improvements in muscle performance with LPLT when combined with physical exercises [46, 47]. However, several other studies have reported improved muscle performance and reduced fatigue as a result of LPLT [47–49]. Lopes-Martins et al. reported that muscle damage and fatigue caused by tetanic contractions in the rat model are seemed to be reduced by LPLT [50]. In the present study, an increase in IPT after the application of HPLT may be due to reduced pain intensity. When pain intensity is reduced, then participants will be able to exert more force on the hamstring muscle.

In the present study, laser therapy was used as a monotherapy because its clinical benefits were reported when used alone [13, 51–54] and also when used in combination with stretching and regular exercises in orthopedic conditions [55, 56]. The clinical implications of the present study include the use of HPLT as an effective treatment modality for athletes with PHT.

The present study also has some limitations. No control group was included in the study where participants did not receive any treatment. Therefore, the reduction in NPRS scores may be due to time travel or avoidance of strenuous activities for 3 weeks and may not be due to the intervention applied. Moreover, the experimental group did not include the conventional physiotherapy treatment; therefore, it cannot be concluded that the improvements observed in the experimental group were additional effects of HPLT. Another limitation is the lack of long-term follow-up. The athletes were not assessed after their return to the sport. It may be possible that the improvement in pain and maximum torque was short-lived. Therefore, future research is needed that includes control group and long-term follow-up. In addition, only male athletes were recruited in the study. Therefore, the results of this study cannot be generalized to female athletes. More research is needed to recruit female athletes with large sample size. Future research should also compare HPLT with LPLT to examine which one is more effective for pain reduction in patients with PHT.

## 4. Conclusions

HPLT was effective in improving pain in athletes with PHT in comparison to conventional physiotherapy program (US, moist heat, and home exercises); however, due to the lack of a control group, the improvement cannot be solely attributed to HPLT. No significant differences were found between HPLT and conventional physiotherapy in improving hamstring IPT, although hamstring IPT increased with HPLT.

## Figures and Tables

**Table 1:** Demographic data, baseline, and postintervention values of outcome variables in both groups (*n* = 18 each group), Shapiro-Wilk test, and independent *t*-test *p* values for baseline values.

	Experimental group	Conventional group	Shapiro-Wilk *p* value	Independent *t*-test *p* values
	Mean ± SD	Mean ± SD		
Age (years)	22.61 ± 1.68	22.39 ± 1.81		
Height (cm)	162.83 ± 9.85	162.22 ± 7.47		
Weight (kg)	58.78 ± 4.91	59.44 ± 3.95		
BMI (kg/m^2^)	22.30 ± 2.58	22.70 ± 2.38		
Pre_NPRS (points)	6.17 ± 1.42	6.61 ± 0.97	0.004^∗^	0.283
Pre_IPT (Nm)	251.17 ± 78.00	236.89 ± 40.34	0.254	0.495
Post_NPRS (points)	2.39 ± 1.03	3.89 ± 0.96		
Post_IPT (Nm)	284.28 ± 109.23	240.44 ± 44.03		

^∗^Significant. SD: standard deviation; BMI: body mass index; NPRS: numeric pain rating scale; IPT: isokinetic peak torque.

**Table 2:** Within-group (Wilcoxon's signed-rank test) and between-group (Mann–Whitney *U* test) comparisons of outcome variables.

	Within-group, *p* values		Between-group, *p* values	
	Experimental group	Conventional group		
Post_NPRS–Pre_NPRS	≤0.001^∗^	≤0.001^∗^	Post_NPRS	≤0.001^∗^
Post_IPT–Pre_IPT	0.028^∗^	0.662	Post_IPT	0.113

^∗^Significant. NPRS: numeric pain rating scale; IPT: isokinetic peak torque.

## Data Availability

The data presented in this study are available in the supplementary material (available here).

## Supplementary Materials

Supplementary Materials Data associated with the study are submitted as supplementary materials.
