# Supplementary material for: Effectiveness of High Power Laser Therapy on Pain and Isokinetic Peak Torque in Athletes with Proximal Hamstring Tendinopathy: A Randomized Trial
Source: Biomed Res Int. 2022 May 20;2022:4133883. doi: 10.1155/2022/4133883 (PMC9142273; doi:10.1155/2022/4133883)
Supplement: Supplementary Materials — Data associated with the study are submitted as supplementary materials. [file 4133883.f1.docx]

| Group | Age (years) | Height (cm) | Weight (Kg) | BMI | Pre_NPRS | Post_NPRS | Pre_IPT | Post_IPT |
| --- | --- | --- | --- | --- | --- | --- | --- | --- |
| 1 | 22 | 162 | 57 | 25.5 | 5 | 2 | 274 | 341 |
| 1 | 24 | 166 | 60 | 21.8 | 7 | 3 | 264 | 308 |
| 1 | 24 | 175 | 61 | 19.9 | 6 | 2 | 233 | 258 |
| 1 | 24 | 155 | 60 | 25 | 4 | 1 | 330 | 408 |
| 1 | 24 | 160 | 62 | 24.2 | 6 | 2 | 279 | 326 |
| 1 | 20 | 156 | 55 | 22.6 | 4 | 2 | 367 | 446 |
| 1 | 20 | 190 | 65 | 18 | 4 | 1 | 279 | 326 |
| 1 | 19 | 156 | 58 | 20.5 | 7 | 3 | 347 | 358 |
| 1 | 21 | 173 | 55 | 21.7 | 8 | 3 | 314 | 322 |
| 1 | 22 | 156 | 55 | 22.6 | 7 | 2 | 279 | 326 |
| 1 | 24 | 160 | 51 | 19.9 | 6 | 3 | 310 | 321 |
| 1 | 22 | 157 | 69 | 28 | 8 | 4 | 251 | 458 |
| 1 | 24 | 155 | 50 | 20.8 | 5 | 2 | 94 | 123 |
| 1 | 23 | 152 | 60 | 26 | 7 | 4 | 180 | 214 |
| 1 | 24 | 155 | 58 | 24.1 | 6 | 1 | 199 | 143 |
| 1 | 24 | 175 | 65 | 21.2 | 8 | 3 | 84 | 93 |
| 1 | 24 | 166 | 62 | 22.5 | 5 | 1 | 193 | 150 |
| 1 | 22 | 162 | 55 | 21 | 8 | 4 | 244 | 196 |
| 2 | 22 | 163 | 58 | 21.8 | 7 | 4 | 221 | 260 |
| 2 | 24 | 168 | 60 | 21.3 | 6 | 4 | 248 | 195 |
| 2 | 24 | 162 | 68 | 25.9 | 8 | 5 | 180 | 188 |
| 2 | 23 | 163 | 60 | 22.6 | 5 | 2 | 229 | 241 |
| 2 | 24 | 165 | 53 | 19.5 | 5 | 3 | 247 | 241 |
| 2 | 19 | 173 | 62 | 20.7 | 8 | 3 | 230 | 299 |
| 2 | 19 | 145 | 57 | 27.1 | 6 | 3 | 250 | 238 |
| 2 | 19 | 164 | 63 | 23.4 | 6 | 4 | 303 | 356 |
| 2 | 21 | 175 | 54 | 17.6 | 8 | 5 | 247 | 241 |
| 2 | 22 | 163 | 57 | 21.5 | 6 | 4 | 203 | 237 |
| 2 | 22 | 163 | 59 | 22.2 | 7 | 4 | 229 | 241 |
| 2 | 23 | 163 | 61 | 23 | 6 | 4 | 231 | 241 |
| 2 | 24 | 163 | 60 | 22.6 | 7 | 5 | 144 | 164 |
| 2 | 23 | 162 | 64 | 24.4 | 7 | 4 | 307 | 297 |
| 2 | 24 | 163 | 56 | 21.1 | 6 | 3 | 214 | 201 |
| 2 | 24 | 145 | 55 | 26.2 | 8 | 6 | 241 | 229 |
| 2 | 24 | 163 | 65 | 24.5 | 6 | 3 | 299 | 230 |
| 2 | 22 | 157 | 58 | 23.5 | 7 | 4 | 241 | 229 |
